# Identification of Inflammation-Related Biomarker Pro-ADM for Male Patients With Gout by Comprehensive Analysis

**DOI:** 10.3389/fimmu.2021.798719

**Published:** 2022-01-18

**Authors:** Kangli Qiu, Tianshu Zeng, Yunfei Liao, Jie Min, Nan Zhang, Miaomiao Peng, Wen Kong, Lu-lu Chen

**Affiliations:** ^1^ Department of Endocrinology, Union Hospital, Tongji Medical College, Huazhong University of Science and Technology, Wuhan, China; ^2^ Hubei Provincial Clinical Research Center for Diabetes and Metabolic Disorders, Wuhan, China

**Keywords:** gout, pro-ADM, comprehensive analysis, inflammation, biomarker

## Abstract

**Objective:**

Gout is a local inflammatory disease caused by the deposition of monosodium urate (MSU) crystals in joints or adjacent tissues. When some gout occurs without hyperuricemia, or its clinical symptoms and signs are not typical, the diagnosis of gout will be delayed, so there is an urgent need to find a new biomarker to predict and diagnose of gout flare. Our research attempts to find the key genes and potential molecular mechanisms of gout through bioinformatics analysis, and collected general data and blood biochemical samples of patients with gout and healthy, then analyzed and compared the expression of factors regulated by key genes.

**Method:**

GSE160170 were downloaded from GEO database for analysis. The data were normalized to identify the differentially expressed genes (DEGs), then GO and KEGG enrichment analysis were applied. Protein-protein interaction (PPI) networks and hub genes between DEGs were identified. Then collect general information and blood samples from male patients with acute gout, hyperuricemia and healthy. ELISA method was used to detect pro-ADM levels of different groups, and the data was input into SPSS statistical software for analysis.

**Result:**

We identified 266 DEGs (179 up-regulated and 87 down-regulated) between gout patients and healthy controls. GO analysis results show that DEGs are mostly enriched in inflammatory response, growth factor activity, cytokine activity, chemokine activity, S100 protein binding and CXCR chemokine receptor binding. KEGG pathway analysis showed that DEGs are mainly related to Chemokine signaling pathway and Cytokine-cytokine receptor interaction. ADM, CXCR1, CXCR6, CXCL3, CCL3, CCL18, CCL3L3, CCL4L1, CD69, CD83, AREG, EREG, B7RP1, HBEGF, NAMPT and S100B are the most important hub genes in the PPI network. We found that the expression of pro-ADM in the gout group and hyperuricemia group was higher than that in the healthy group, and the difference was statistically significant.

**Conclusion:**

In this study, a series of bioinformatics analyses were performed on DEGs to identify key genes and pathways related to gout. Through clinical verification, we found that pro-ADM can be used as an inflammation-related biomarker for acute attacks of gout, providing new ideas for the diagnosis and treatment of gout.

## Introduction

Gout is a local inflammatory disease caused by the deposition of monosodium urate (MSU) crystals in joints or adjacent tissues. The pain is often described as burning, tingling or biting (1). Clinically, it can be manifested as gouty arthritis, tophi, uric acid kidney stones or gouty nephropathy. According to data reported in different studies, the global incidence rate is between 0.6-2.9/1000 person-years, and the prevalence rate is between 0.68%-3.90% in adult (2, 3). The incidence and prevalence of gout increase with age, and it is more common in men than in women. In Asia, the sex ratio of gout is about 8:1, which is much higher than in Europe and America (4–6).

The risk factors of gout include both genetic and non-genetic. Among them, hyperuricemia is the most important risk factor for the gout flare. There is a concentration-dependent relationship between serum uric acid levels and the risk of gout (7). Factors which lead to elevated uric acid are also risk factors for gout, such as alcohol, red meat, seafood, sugary drinks, diuretics and chronic kidney disease (8–10). For genetics part, 55 loci have been determined to be related to the risk of gout in the genome-wide association study (11).

The gold standard for diagnosis of gout is the presence of MSU crystals in synovial fluid or tophi under the microscope. MSU crystals are damage-related molecules that stimulate the innate immune pathway. In the pathogenesis of gout, NLRP3 inflammasome is the main way for MSU crystals to trigger cellular inflammatory response. Inflammatory cytokines, especially IL-1β, are key mediators of gout inflammation (12). A variety of regulatory pathways have been found to regulate the activity of inflammasomes and the release of IL-1β (12–15). Many studies have confirmed that the expression levels of some inflammatory factors, including IL-1β, IL-6, IL-8 and TNF-α, are significantly increased in patients with acute gout flare (16, 17), while α1­antitrypsin (AAT) or some anti- Inflammatory factors such as IL-37, TGF-β, IL-10 and IL-1RA (IL1RN) are negative regulators of gout inflammation (18–21). The study by Yu Wang et al. confirmed that the serum levels of xanthine and hypoxanthine in patients with gout were significantly increased, xanthine and hypoxanthine have clinical application value in the diagnosis of gout especially in patients with normal uric acid (22). Xueshan Bai et al. found that serum CA72-4 levels are elevated in patients with frequent attacks of gout and can be used as a predictor of gout attacks (23). However, the biomarkers related to gout inflammation are still unknown, which limits the prediction of gout flare and makes atypical gout misdiagnosed or delayed in diagnosis. The basic research on the pathogenesis of gout and clinical diagnosis and treatment are still in continuous progress and exploration. Our research attempts to find the key genes and potential molecular mechanisms of gout through bioinformatics analysis, and then collect general data and blood biochemical samples of gout patients and normal patients, analyze and compare the expression of key gene regulatory factors, and verify it in the clinic, finally providive a basis for finding novel biomarkers of gout.

## Materials and Methods

### Data Selection

The GSE160170 data set is downloaded from the GEO official website, and the expression matrix uses GPL21827 [HuGene-1_0-st] Affymetrix human gene 1.0 ST array [transcript (gene) version]. The data set includes six gout samples and six normal samples.

### Data Processing

Evaluating the GSE160170 raw data set by using the limma R package. Our study first corrected the data, obtained the expression matrix data set required by the experiment by taking the form of a subset, and then extracted the clinical information of the corresponding sample according to the data sample of the expression matrix for subsequent sample classification, and finally performed the data on GSE160170 according to the gene ID. The normalization process eliminates the influence caused by the batch effect. Through data processing, we finally obtained normal samples (6 cases) as the control group and gout samples (6 cases) as the treat group, using |log2 FC|>2 and adjusted p<0.05 to identify gout-deg.

### Enrichment Analysis

The database uses the org.Hs.eg.db database file on the bioconductor platform. The file contains 28 mainstream data files. We analyze the differential genes of gout. GO enrichment analysis (24) and KEGG pathway enrichment analysis (25) analyze the biological processes and key pathways that differential genes are mainly involved in. Among them, GO enrichment analysis P<0.01 is the selection criterion, and KEGG pathway analysis is based on P<0.05 for selection criteria.

### Construction of PPI Network

The search tool for searching interacting genes (STRINGv-11.0, https://string-db.org/) database is an online tool for evaluating PPI information. To evaluate the interaction relationship between DEGs, DEGs are mapped to STRING, and the interaction relationship between DEGs is screened from the protein level, and a PPI network that up-regulates and down-regulates DEGs is constructed. Then we used Cytoscape software to construct PPI network visualization. The cytohHubba plug-in was used to screen the HUB genes of the PPI network in Cytoscape, and TOP25 HUB genes were selected for analysis by using Density of Maximum Neighborhood Component (DMNC) in the local-based method and EcCentricity (EC) in the global-based method respectively.

### Clinical Patient Selection

This study included 4 patients with acute gout, 6 patients with asymptomatic hyperuricemia (HUA) (no clinical manifestations of gouts, erum uric acid ≥428mol/L), and 4 healthy people (serum uric acid <428mol/L), These patients were all male and selected from the Department of Endocrinology, Union Hospital, Tongji Medical College, Huazhong University of Science and Technology. The exclusion criteria are: 1. Patients with Cushing syndrome and patients who have previously used glucocorticoids and non-steroidal anti-inflammatory drugs; 2. Patients suffering from diabetes, hypertension, tumors, liver and kidney insufficiency, acute or chronic infectious diseases, immune system diseases, cardiovascular diseases.

### Enzyme-Linked Immunosorbent Assay (ELISA)

Pro-ADM levels were determined by using the human Pro-ADM ELISA kit (CSB-E14356h, CUSABIO, Wuhan, China) according to the manufacturer’s instructions.

### Statistical Analysis

The clinical data was analyzed by SPSS28.0 software (IBM, Armonk, NY, USA). The measurement data is represented by the mean ± standard deviation. A one-way analysis of variance (ANOVA) was used to assess the differences between the three groups. P value<0.05 is considered statistically significant.

## Results

### Identification of Differentially Expressed Genes for Gout

**Figure 1:**
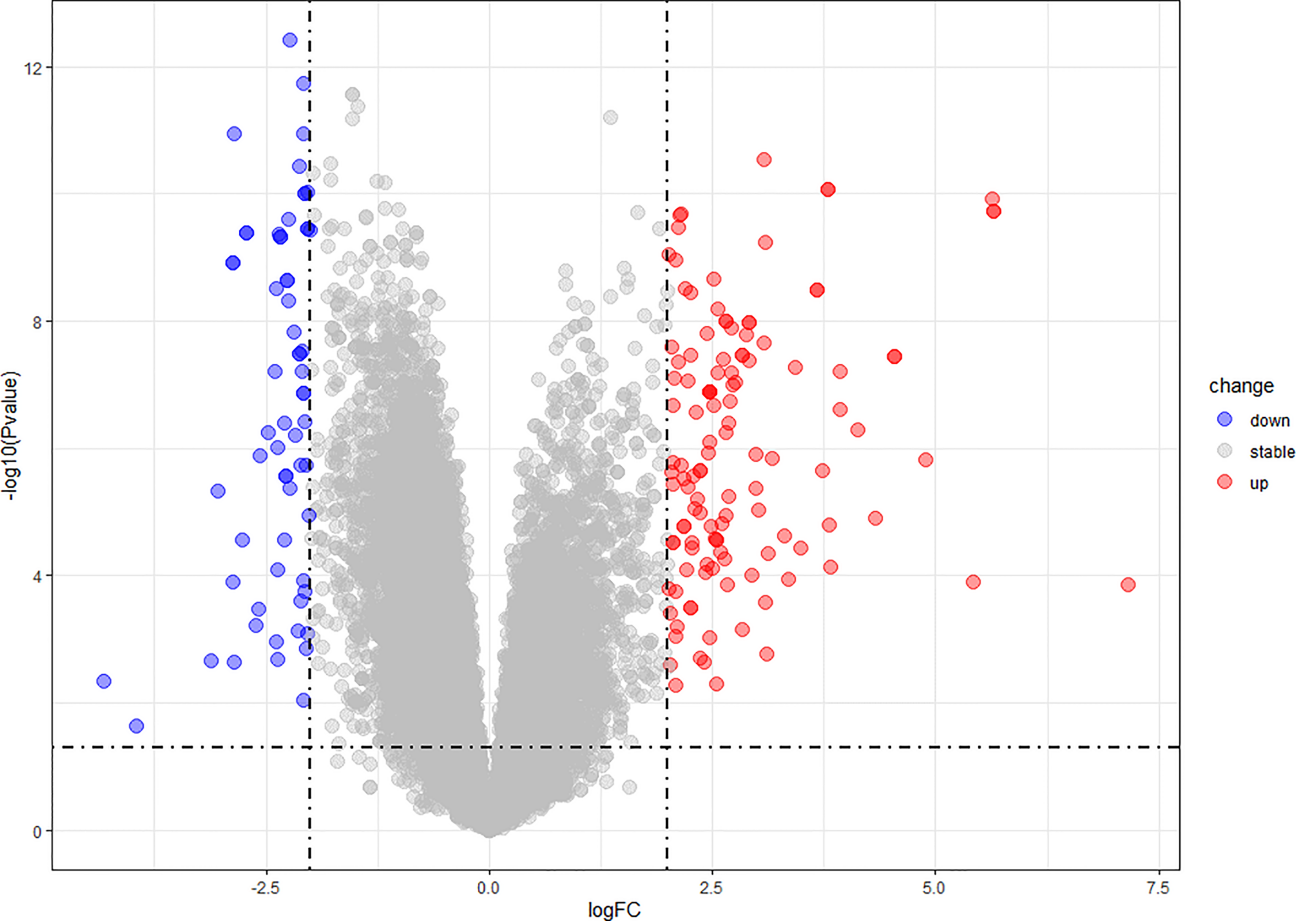
Volcano map of DEGs.

**Figure 2:**
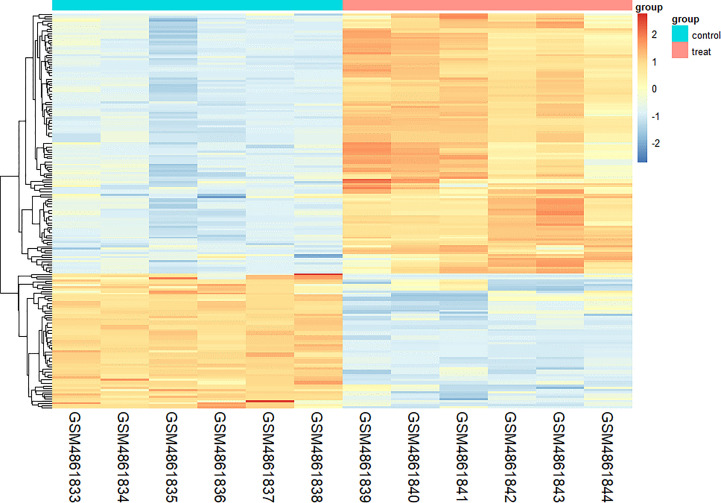
Heat map of DEGs.

We found 266 DEGs in gout patients, including 179 up-regulated genes and 87 down-regulated genes compared with the healthy control group. We drew a volcano map (**Figure 1**) and a hierarchical clustering heat map of differential genes (**Figure 2**). The results show that there is a good difference between these DEGs between the gout group and the control group. PTPRS and DCLRE1C were identified as the most up-regulated and down-regulated genes in gout patients respectively.

### Functional Enrichment Analysis

**Figure 3:**
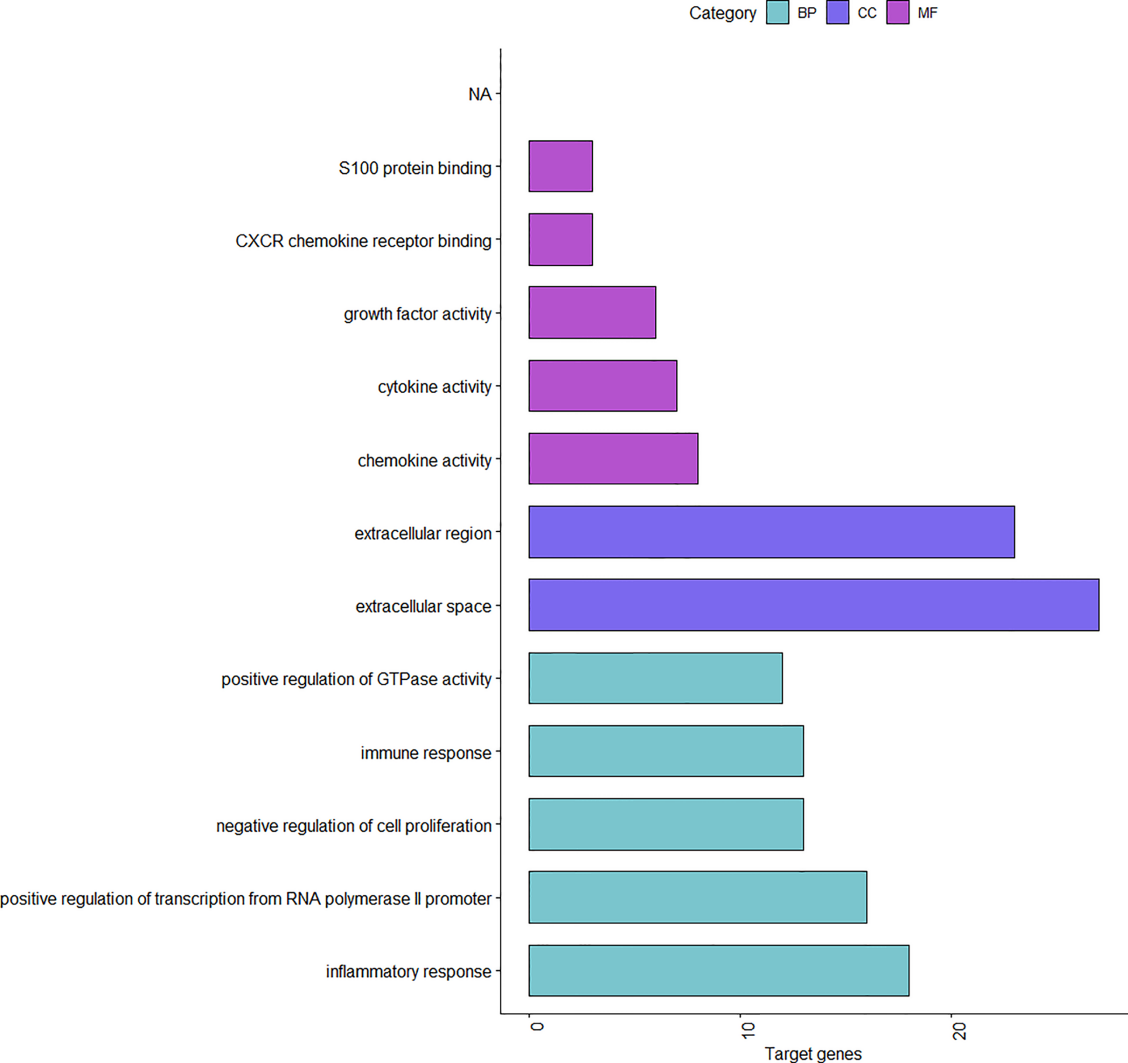
The results of GO of DEGs.

**Figure 4:**
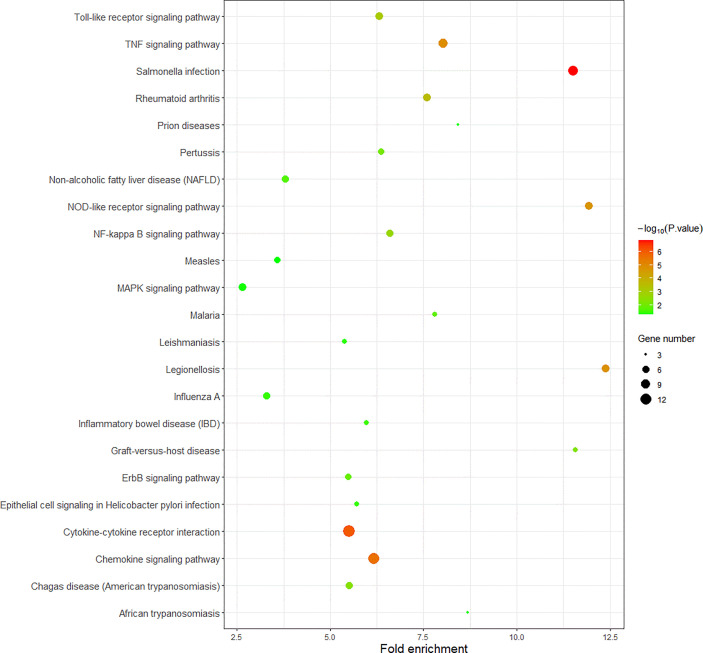
The results of KEGG of DEGs.

We use R language to perform GO analysis of differential genes by using the org.Hs.eg.db database file on the bioconductor platform and use David (https://david.ncifcrf.gov/) online tools to perform KEGG enrichment analysis. For biological processes, molecular function and cell composition, a total of 12 GO (**Figure 3** and **Table 1**) and 23 KEGG pathways (**Figure 4**) were identified. BP mainly focuses on inflammatory response, negative regulation of cell proliferation, immune response,positive regulation of transcription from RNA polymerase II promoter and positive regulation of GTPase activity, CC mainly focuses on extracellular space and extracellular region, MF mainly focuses on chemokine activity and cytokine activity, Growth factor activity, CXCR chemokine receptor binding and S100 protein binding. In addition, KEGG pathway analysis showed that DEGs are closely related to Salmonella infection, Chemokine signaling pathway and Cytokine-cytokine receptor interactio. We can conclude that ADM is located in the extracellular region and region, and participates in the negative regulation of cell proliferation.

**Table 1:** The results of GO of DEGs.

Category	Term	Count	PValue	Genes
BP	inflammatory response	18	6.43E-09	CXCL8, CCL3L1, CCL4L2, TNFAIP3, CXCL1, CXCR6, LYZ, CXCL3, PTGS2, CXCL2, TNF, IL1A, IL6, CXCR1, IL1B, NFKBIZ, CCL3, CCL18
BP	positive regulation of transcription from RNA polymerase II promoter	16	0.009519	CSRNP1, LUM, NRG1, AATF, TNF, CDC73, NR4A2, IL1A, NR4A1, IL6, NR4A3, IL1B, NAMPT, MAFK, OGT, ATF3
BP	negative regulation of cell proliferation	13	6.36E-05	BTG1, CXCL8, CCL3L1, ADM, CXCL1, PTGS2, CDC73, EREG, IL1A, IL6, ADAMTS1, IL1B, FKTN
BP	immune response	13	0.000114	CXCL8, CCL4L2, CXCL1, CXCL3, SLED1, CXCL2, TNF, IL1A, IL6, RGS1, IL1B, CCL3, CCL18
BP	positive regulation of GTPase activity	12	0.004734	FARP1, PLEKHG1, ARHGEF9, CCL3L1, ADGRB3, RGS1, CCL4L2, CCL3, NRG1, CCL18, EREG, HBEGF
CC	extracellular space	27	3.06E-06	CXCL8, CCL3L1, CCL4L2, ADM, CXCL1, CXCL3, TNF, CXCL2, AREG, GPC1, NAMPT, CCL3, CCL18, PTGDS, FKTN, SLF2, EDN2, LUM, NRG1, S100B, LYZ, EREG, IL1A, IL6, IL1B, EZR, HBEGF
CC	extracellular region	23	0.002473	EDN2, CXCL8, OVCH1, CCL3L1, LUM, SPINK6, CCL4L2, ADM, CXCL1, NRG1, LYZ, CXCL3, S100B, C14ORF93, CXCL2, TNF, EREG, IL1A, IL6, IL1B, CCL3, PTGDS, HBEGF
MF	chemokine activity	8	8.05E-08	CXCL8, CCL3L1, CCL4L2, CCL3, CXCL1, CXCL3, CCL18, CXCL2
MF	cytokine activity	7	0.002241	IL1A, IL6, IL1B, NAMPT, NRG1, TNF, AREG
MF	growth factor activity	6	0.00786	IL6, CXCL1, NRG1, AREG, EREG, HBEGF
MF	CXCR chemokine receptor binding	3	0.001983	CXCL1, CXCL3, CXCL2
MF	S100 protein binding	3	0.004213	S100A1, EZR, S100B

### PPI Network and Hub Gene Identification

**Figure 5:**
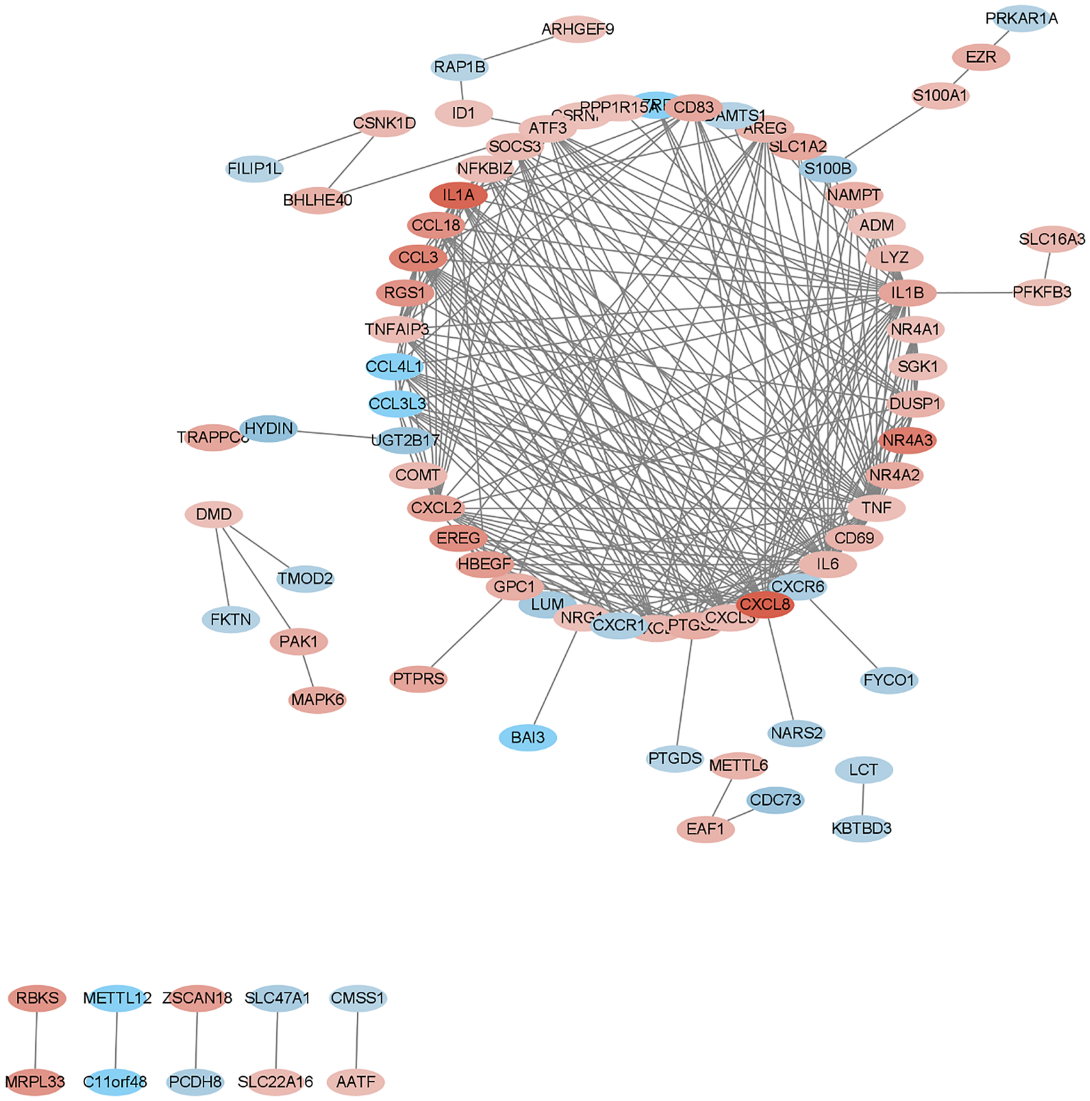
PPI network of the DEGs.

**Figure 6:**
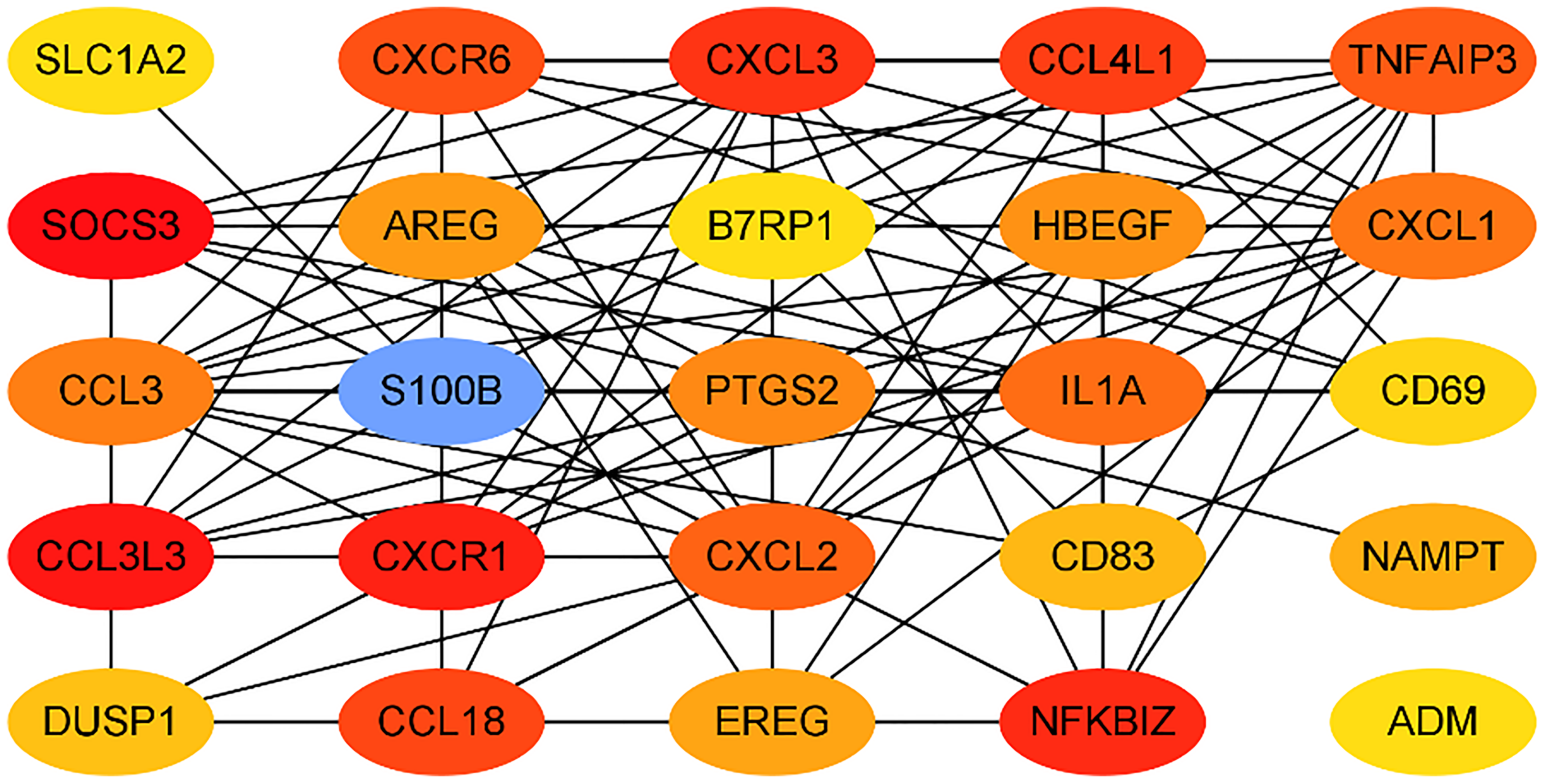
Hub genes in the PPI network by DMNC.

**Figure 7:**
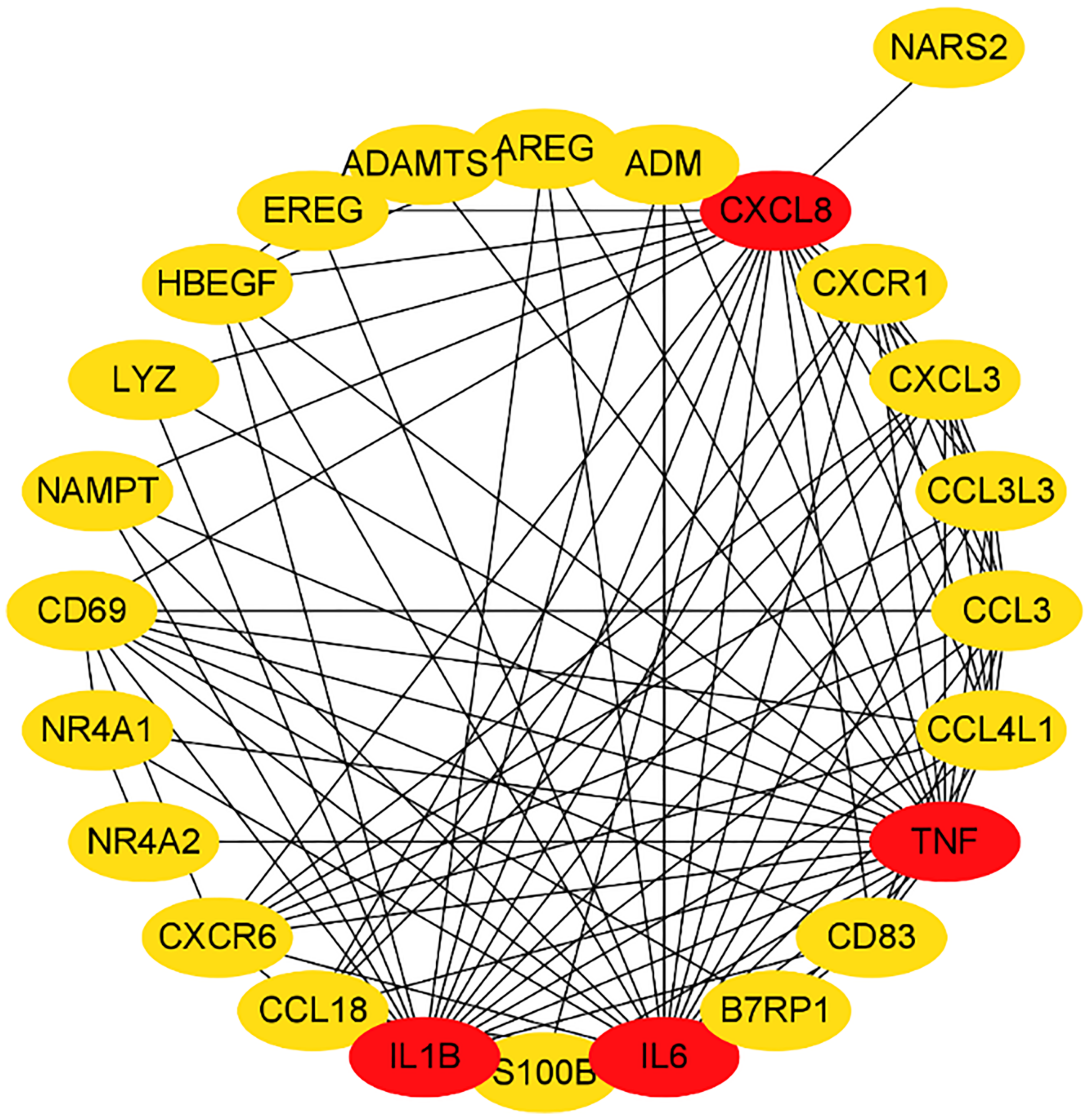
Hub genes in the PPI network by EC.

We submitted DEGs to STRING database to obtain PPI data, and used Cytoscape 3.8.2 to construct a PPI network (**Figure 5**), and used two algorithms to determine the top 25 genes as key genes (**Figures 6** and **7**). We intersect the key genes of the two algorithms to obtain a total of 16 key genes, namely ADM, CXCR1, CXCR6, CXCL3, CCL3, CCL18, CCL3L3, CCL4L1, CD69, CD83, AREG, EREG, B7RP1, HBEGF, NAMPT and S100B.

### Verification of Pro-ADM in Clinical Samples

Through bioinformatics methods, we found that ADM is not only an up-regulation of DEG for gout, but also highly correlated with pathways obtained by differential gene enrichment analysis, and it is also one of the hub genes in the PPI network of the DEGs. In order to verify the clinical application potential of ADM gene, ELISA method was used to detect the protein level encoded by ADM gene in clinical samples. ADM is a peptide hormone isolated from human adrenal medulla chromaffin cells and belongs to the calcitonin gene-related peptide superfamily. Due to the short half-life of ADM, pro-adrenomedullin (pro-ADM) detection is used clinically instead.

**Figure 8:**
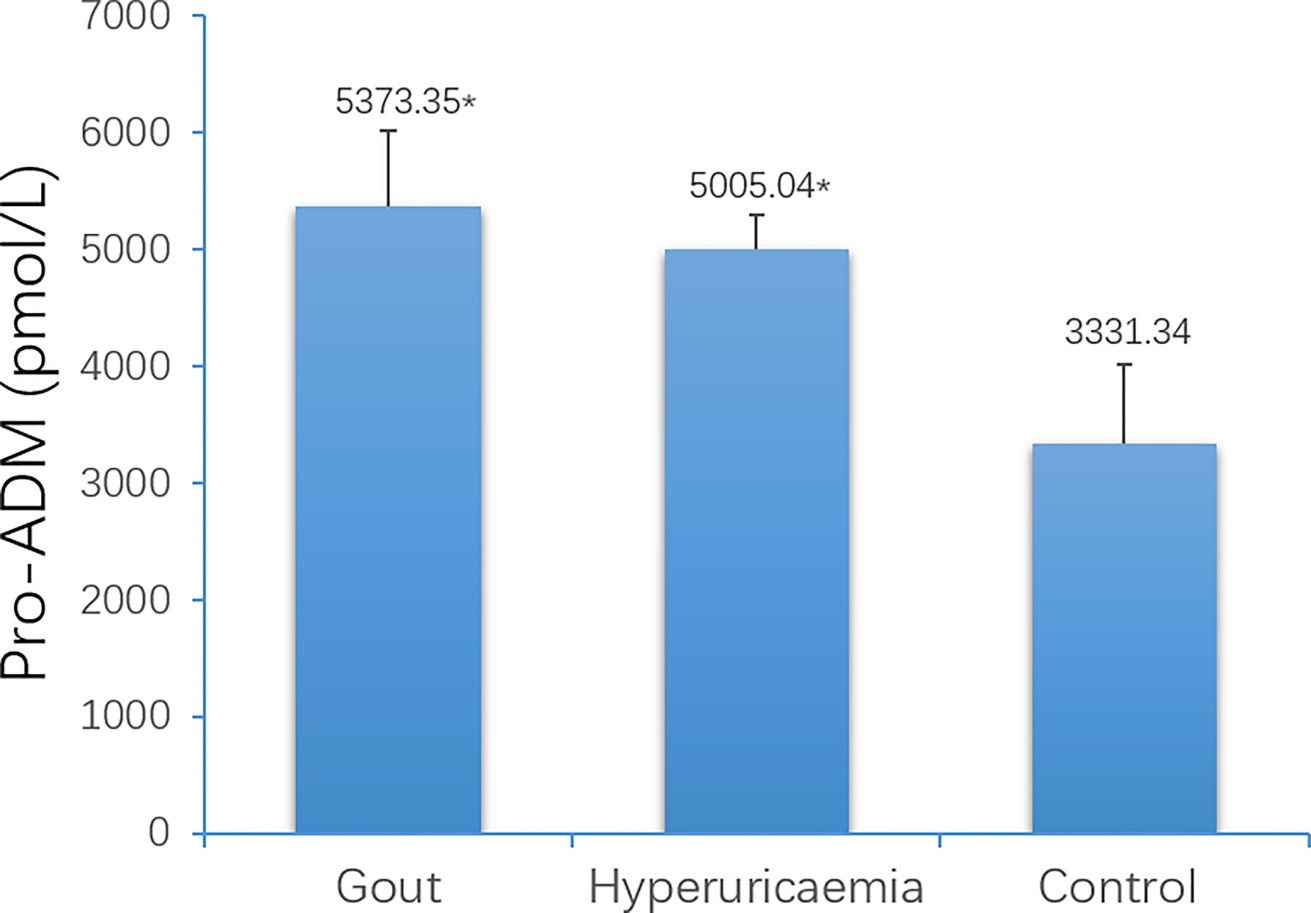
Comparison of serum Pro-ADM levels among gout patients, hyperuricaemia patients and control (*P < 0.05 vs. control).

The average age of the three groups was: 34 years old in the gout group, 35 years old in the HUA group, and 44 years old in the healthy control group. There was no significant difference in age (P> 0.05) (**Table 2**). The blood uric acid level of the gout group and the hyperuricemia group was higher than that of the control group, and the difference was statistically significant (**Table 2**). The expression of serum pro-ADM of the acute gout group and the hyperuricemia group was higher than that of the control group, and the difference was statistically significant (**Figure 8**).

**Table 2:** Comparison of datas of patients in three groups (*P < 0.05 vs. control).

Indicator (median)	Gout (n = 4)	Hyperuricaemia (n = 6)	Control (n = 4)
Age (years)	34	35	44
Uric acid (μmol/L)	512.08*	505.8*	361.7
Pro-ADM (pmol/L)	5373.35 ± 1291.41*	5005.04 ± 691.23*	3331.3 ± 1370.85

## Discussion

Gout is a metabolic disease that seriously endangers human health. It is caused by local inflammation caused by the deposition of monosodium urate (MSU) crystals in joints or adjacent tissues. In this study, we downloaded and analyzed data from 6 gout patients and 6 healthy controls from the GEO database. We identified a total of 266 DEGs, including 179 up-regulated DEGs and 87 down-regulated DEGs. These genes include PTPRS, SLED1, ACSL1, CXCL8, DCLRE1C, FAM217A and ADM, etc. Then we use the database to enrich and analyze these differential genes. The results show that these genes are mainly involved in immune response, inflammatory response and other processes, and are enriched in chemokine signaling pathways and cytokine-cytokine receptor interactions. Process-related genes include CXCL8, CCL3L1, ADM, CXCL1, TNF, IL1A, IL6, etc., among which ADM is highly related to these pathways. Finally, by constructing a PPI network, we took the intersection of the hub genes obtained by two algorithms, and obtained a total of 16 key genes, namely CXCR1, CXCR6, CXCL3, CCL3, CCL18, CCL3L3, CCL4L1, CD69, CD83, AREG, EREG, B7RP1, HBEGF, NAMPT, S100B and ADM.

We analyzed these 16 genes in the next step. CXCR1, CXCR6, CXCL3, CCL3, CCL18, CCL3L3, CCL4L1 are all family members related to chemokines. The protein encoded by the CXCR1 gene is a member of the G protein-coupled receptor family and is a receptor for interleukin 8 (IL8). The combination of two leads to the activation of neutrophils (26). CXCR1 related diseases include acute pyelonephritis, cancers and human immunodeficiency virus type 1 (27), etc. related pathways include Akt signaling pathway and CCR5 pathway in macrophages. Yangang Wang et al. conducted a case-control study to investigate the relationship between the onset of gouty arthritis in Chinese Han men with CXCR1 and CXCR2 gene polymorphisms. The study found that the CXCR1 gene rs2234671 and CXCR2 gene rs1126579 in the gouty arthritis group were not related to the susceptibility of gout in Chinese men. The CXCR2 gene rs2230054 is related to the susceptibility of gout in Chinese men (28). Research by Ying Ye et al. showed that CXCR1/CXCR2 antagonist G31P can reduce the inflammatory progression of chronic uric acid nephropathy and play a role in renal protection (29). CXCR6’s exclusive ligand Chemokine Ligand 16 (CCL16) is a part of the signaling pathway that regulates the migration of T lymphocytes to various peripheral tissues (lung, intestine, liver, skin and spleen red pulp), and promotes cell-cell migration. The diseases associated with CXCR6 include tumors, diabetes, respiratory diseases and immunodeficiency (30–32). Studies have found that the concentration of CXCL16 is significantly increased in the synovial fluid of patients with gout, and the migration of polymorphonuclear neutrophils in response to CXCL16 has been observed *in vitro* (33). CCL3 encodes macrophage inflammatory protein 1 α (M1P-1 α), which plays a role in inflammation by binding to CCR receptors. Related pathways include Akt signaling pathway and CCR5 pathway in macrophages (34). Studies have shown that the combination of TNF-α, GM-CSF and MSU will cause neutrophils to produce IL-8 and eliminate the release of MIP-1α, leading to the recruitment of neutrophils, which is consistent with the pathological state of gout (35). The expression of CD69-encoded protein is induced when T lymphocytes are activated, and may play a role in proliferation. Studies have found that the expression of CD69 in mucosal-associated invariant T (MAIT) cells in gout patients is increased, and it is increased by the stimulation of MSU crystals (36), and studies have suggested that allopurinol, a gout treatment drug, can attenuate the upregulation of CD69 (37)..

ADM is a peptide hormone isolated from human adrenal medulla chromaffin cells and belongs to the calcitonin gene-related peptide superfamily. ADM is mainly synthesized and secreted by vascular endothelial cells and smooth muscle cells, and mRNA is highly expressed in adrenal gland, heart, lung, kidney and other tissues. ADM can bind to CGRP receptors or its specific receptors, and exert various physiological effects through nitric oxide (NO), cyclic adenosine monophosphate (cAMP), IP3-Ca2+ or cyclic guanosine monophosphate (cGMP) pathways. ADM has a wide range of physiological effects. It has the effects of inhibiting the secretion of aldosterone, natriuretic and diuretic, inhibiting the proliferation of vascular smooth muscle, and anti-infection. ADM is closely related to diseases such as heart failure, myocardial infarction, sepsis. The plasma ADM can not only be used for the treatment of heart failure, but its elevated index is also an independent factor for the poor prognosis of chronic congestive heart failure (38). ADM can be used as an independent indicator of the prognosis of acute myocardial infarction (39). ADM can also help diagnose sepsis and assess its severity. Because ADM has a short half-life, clinically, the intermediate products that are stable when ADM is cleared in the circulation, namely adrenomedullin precursor (pro-ADM) and intermediate adrenomedullin precursor (MRpro-ADM) are used instead. ADM is generated from the ADM precursor consisting of 185 amino acids (preproadrenomedullin) by post-translational enzymatic processing (40). ADM is processed from proadrenomedullin as glycine-extended ADM (ADM-glycine), an intermediate form of ADM. Subsequently, mature ADM is converted from ADM-glycine by enzymatic amidation (41). Another 20-amino acid peptide is also generated and called proadrenomedullin N-terminal 20 peptide (PAMP) (42, 43).The mechanisms of the increase of pro-ADM in different diseases are not completely the same, but they are all related to the physiological effects of ADM. The increase in blood volume and activation of sympathetic nerves in patients with chronic heart failure leads to an increase in plasma ADM concentration. The increased secretion of ADM can dilate blood vessels, maintain vascular integrity, inhibit the renin-angiotensin-aldosterone system, and inhibit ventricular remodeling (44). The increase in ADM in patients with infectious diseases such as sepsis is due to its bactericidal, anti-inflammatory, and immune-regulating effects (45). In addition to its own antibacterial and anti-infective effects, ADM can also bind to complement regulatory factor H and interact with it. Complement regulatory factor H prolongs the action time of ADM, and ADM accelerates the clearing of C3b (46).

As an emerging indicator of inflammation, pro-ADM has not been studied to show changes in patients with gout. We speculate that the involvement of ADM in gout may be related to the following aspects. Immune cells such as macrophages, lymphocytes, neutrophils and microglia in the body can synthesize and secrete ADM. ADM secreted by immune cells can inhibit the up-regulation of neutrophil CD11b levels and increase the content of neutrophil cAMP. Under the stimulation of bacterial mucopolysaccharide, ADM can increase IL-6 and decrease the secretion of TNF-α. Macrophages are important innate immune cells in the human body. They differentiate into different phenotypes at different stages of gout, and participate in the occurrence and alleviation of inflammation. Studies have shown that monocytes/macrophages should be considered as the main source of ADM in the circulating blood, and the secreted ADM may regulate the function of macrophages (47). In addition, TNF-α and IL-1β can enhance the synthesis and secretion of ADM (48, 49). We already know that the key step of gout attacks also include neutrophil activation leading to apoptosis inhibition and degranulation. ADM can inhibit mitochondrial-mediated apoptosis through the Akt/GSk-3β pathway, reducing the activity of caspase3, cytochrome C translocation from mitochondria to Cytoplasmic is inhibited, the mRNA and protein expression of Bcl-2 increases, and the Bcl-2/Bax ratio increases. This may also be one of the ways ADM participates in the regulation of gout. When gout affects the kidneys in the late stage, renal insufficiency and even acute and chronic renal failure may occur, manifested by symptoms such as water and sodium retention, high blood pressure, and heart failure. These symptoms can lead to increased blood volume and activation of sympathetic nerves, thereby promoting the secretion of ADM and the increase of pro- ADM levels. This theoretical speculation is consistent with the conclusion we have reached through bioinformatics methods: ADM is not only an up-regulation of DEG for gout, but also highly correlated with pathways obtained by differential gene enrichment analysis, and it is also one of the hub genes in the PPI network. Finally, through the detection of pro-ADM levels in clinical samples, we confirmed that pro-ADM is involved in gout flare. Patients with gout and hyperuricemia are relatively easy to distinguish by clinical symptoms and signs. The difficulty in clinical diagnosis of gout is that the blood uric acid level of gout patients is not necessarily elevated, so it is difficult to distinguish it from other arthralgia diseases. We also conducted DEGs analysis on rheumatoid arthritis and spondyloarthritis through bioinformatics methods. GSE134087 (rheumatoid arthritis) and GSE58667 (spondy arthritis) were downloaded from GEO database. We found that ADM is a stably expressed gene for rheumatoid arthritis and spondyloarthritis. Therefore, we believe that detecting the level of Pro-ADM is helpful for diagnosing gout, which is also the clinical significance of this study.

The main limitations of this study are that the sample size is small and all the participants are male, which may lead to the non-universal results. In future work, we will further expand the sample size and include female investigators in the study. In addition to gout, pro-ADM may also be affected by other factors. Finally, all subjects in this study are Chinese, and it is uncertain whether the results of this study can be generalized to other races. Despite these limitations, this is still the first one to study the potential biomarkers and pathogenesis of the acute attacks of gout through bioinformatics methods combined with clinical sample verification.

## Conclusions

Our data provides a comprehensive DEGs bioinformatics analysis to find molecular mechanisms related to gout. We found that pro-ADM can be used as a new inflammation-related biomarker to predict and diagnose the acute attacks of gout in male patients, which provides new insights for the development of it. In the future, further experiments at the cellular and molecular levels will be needed to confirm its role in pathogenesis of gout.

## Data Availability Statement

The original contributions presented in the study are included in the article/supplementary material. Further inquiries can be directed to the corresponding authors.

## Ethics Statement

The studies involving human participants were reviewed and approved by Medical Ethics Committee of Union Hospital, Tongji Medical College, Huazhong University of Science and Technology. The patients/participants provided their written informed consent to participate in this study.

## Author Contributions

KQ: Conceptualization, Methodology, Validation, Data curation, Writing- Original draft preparation. TZ: Conceptualization, Methodology, Supervision, Writing - Review & Editing. YL: Resources, Visualization, Validation. JM: Resources, Visualization, Software. NZ: Data Curation, Formal analysis, Resources. MP: Data Curation, Formal analysis, Investigation. WK: Funding acquisition, Writing- Reviewing and Editing. L-lC: Funding acquisition, Writing- Reviewing and Editing. All authors contributed to the article and approved the submitted version.

## Conflict of Interest

The authors declare that the research was conducted in the absence of any commercial or financial relationships that could be construed as a potential conflict of interest.

## Publisher’s Note

All claims expressed in this article are solely those of the authors and do not necessarily represent those of their affiliated organizations, or those of the publisher, the editors and the reviewers. Any product that may be evaluated in this article, or claim that may be made by its manufacturer, is not guaranteed or endorsed by the publisher.
